# A High-Sensitivity Bowel Sound Electronic Monitor Based on Piezoelectric Micromachined Ultrasonic Transducers

**DOI:** 10.3390/mi13122221

**Published:** 2022-12-14

**Authors:** Xiaoxia Ding, Zhipeng Wu, Mingze Gao, Minkan Chen, Jiawei Li, Tao Wu, Liang Lou

**Affiliations:** 1 School of Microelectronics, Shanghai University, Shanghai 201800, China; 2 The Shanghai Industrial μTechnology Research Institute, Shanghai 201899, China; 3 School of Information Science and Technology, ShanghaiTech University, Shanghai 201210, China

**Keywords:** PMUTs, bowel sounds, health monitoring, high sensitivity

## Abstract

Bowel sounds contain some important human physiological parameters which can reflect information about intestinal function. In this work, in order to realize real-time monitoring of bowel sounds, a portable and wearable bowel sound electronic monitor based on piezoelectric micromachined ultrasonic transducers (PMUTs) is proposed. This prototype consists of a sensing module to collect bowel sounds and a GUI (graphical user interface) based on LabVIEW to display real-time bowel sound signals. The sensing module is composed of four PMUTs connected in parallel and a signal conditioning circuit. The sensitivity, noise resolution, and non-linearity of the bowel sound monitor are measured in this work. The result indicates that the designed prototype has high sensitivity (−142.69 dB), high noise resolution (50 dB at 100 Hz), and small non-linearity. To demonstrate the characteristic of the designed electronic monitor, continuous bowel sound monitoring is performed using the electronic monitor and a stethoscope on a healthy human before and after a meal. Through comparing the experimental results and analyzing the signals in the time domain and frequency domain, this bowel sound monitor is demonstrated to record bowel sounds from the human intestine. This work displays the potential of the sensor for the daily monitoring of bowel sounds.

## 1. Introduction

In recent years, intestinal diseases have become a multi-morbidity threat to human health. Several studies [1,2,3,4] have shown that bowel sounds (BS) can reflect the information about intestinal function and are an important indicator to assist physicians in diagnosing intestinal diseases and judging patients’ recovery after surgery. Human intestinal activities cause the movement of the intestinal contents, during which the bowel sounds are produced [5]. Intestinal activities in different pathological states can cause the bowel sounds to have different characteristics [6,7]. Bowel sounds are weak and irregular, which make their diagnosis very difficult and fail to bring out their clinical value. Currently, physicians mainly use a stethoscope to hear bowel sounds. This method allows for the recording and digitization of bowel sounds. In addition, people are now becoming more and more aware of their gastrointestinal health, and stethoscopes cannot meet the demands of home health monitoring. Therefore, sensors for collecting bowel sounds are required to be portable, wearable, and highly sensitive.

In the existing studies of bowel sounds, several solutions have been proposed to acquire the bowel sounds [8,9,10,11,12,13,14,15,16,17,18,19,20,21,22]. Kolle et al. [13] used a condenser microphone to record bowel sounds in the right upper quadrant of the abdomen for meal detection. Qiao et al. [17] proposed a dual-channel, stethoscope-enhanced microphone (adding a microphone to the head of the stethoscope) to capture the bowel sounds of the human body and extract the time–domain and frequency–domain features of the signals. The designed system was able to achieve a detection accuracy of 85.7%. Horiyama et al. [21] applied a commercial electronic stethoscope to capture the bowel sounds of increased gas in the gastrointestinal tract due to soda ingestion. Du et al. [22] designed a simple piezo-acoustic sensing device for long time bowel sound recording. Among them, microphones are susceptible to interference noise and are easily limited by environmental conditions such as speaking voices. Additionally, the modified stethoscope needs to rely on the head of the stethoscope, which may cause discomfort (cold feeling) when attached to the skin and has a large size, and the commercial electronic stethoscope is expensive, resulting in high cost.

Micro-electro-mechanical systems (MEMS) have been rapidly developed in recent years. MEMS technology allows a large amount of compact-sized devices to be produced at a low cost with high performance, which makes it useful in many applications, such as medical imaging, object recognition, and rangefinding [23,24,25,26,27,28]. Moreover, MEMS technology has been applied to the production of PMN-PT composite material and the development of high frequency ultrasonic arrays [29,30,31]. The main advantages of MEMS sensors include compact size, low power consumption, easy integration, and so on. It is of interest that MEMS microphones (silicon microphones) have been used for bowel sound monitoring in some recent studies [32,33,34,35,36] due to their small size and high sensitivity. Wang et al. [32] developed a flexible, wearable wireless system using silicon microphone, which could realize long-term, real-time monitoring and analysis of bowel sounds. The designed bowel sound monitoring device tended to cause discomfort to the human body because it had to be fully attached to the abdominal wall. O. Sakata’s team [33] designed a simple sensor using MEMS microphones to evaluate the effect of frictional sounds generated by clothing on the detection of bowel sounds in a moving patient. The designed sensor was unwearable and did not facilitate real-time monitoring. Baronetto et al. [36] embedded an array with eight MEMS microphones on a T-shirt connected to a wearable computer for capturing bowel sounds generated during digestion. The designed smart T-shirt had some limitations due to the different body sizes of people. In short, the current research on bowel sound sensors cannot simultaneously balance wearability, portability, and miniaturization. Piezoelectric micromachined ultrasonic transducers (PMUTs) are smaller in size and higher in frequency than MEMS microphones, allowing for smaller packages and detection of a larger frequency range.

This work puts forward a bowel sound electronic monitor based on PMUTs, aiming to providing long-time and real-time monitoring of bowel sounds. This prototype has high sensitivity of −142.69 dB and noise resolution of 50 dB (at 100 Hz) and is composed of a sensing module and a GUI (graphical user interface) based LabVIEW. The sensing module consists of four connected-parallel PMUTs and a signal conditioning circuit. This work designed a GUI based LabVIEW for displaying and storing bowel sounds through the data acquisition card. Continuous bowel sound monitoring is performed using the bowel sound monitor and a stethoscope on a healthy adult male before and after a meal. Experimental results demonstrate its effectiveness in long-time continuous monitoring of bowel sounds during digestion. What is more, the reported bowel sound monitor has compact size and wearability features and possesses good potential in daily health monitoring.

## 2. Structure and Characteristics of the PMUT Array

### 2.1. Structure of the PMUT Array

**Figure 1:**
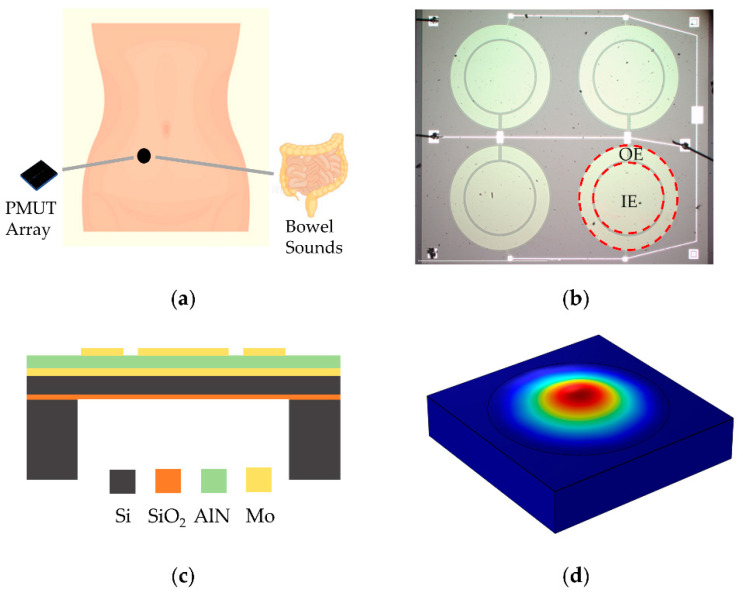
The PMUT array-based acoustic sensor for monitoring bowel sounds. (**a**) The conceptual representation of intestinal auscultation to simultaneously monitor bowel sounds. (**b**) Optical image of the fabricated PMUT array. (**c**) Schematic cross-sectional view of a sensing cell in the PMUT array. (**d**) Simulated mode shape of a sensing cell in the PMUT array.

The application scene of the bowel sound monitor is shown in Figure 1a. Bowel sounds are produced by the intestine pushing the intestinal contents and are spread through the intestinal wall to the abdominal wall [37]. Therefore, placing the sensor on the human abdomen is a direct and effective method. These vibration signals can be detected by the PMUTs. Then, the diaphragm of the PMUTs bend as the bowel sound changes. The bending deflection of the PMUTs sensing diaphragm produces a transverse stress and generates electrical charges [38]. As a result, the PMUTs complete the acoustic-electric conversion.

Commonly used piezoelectric materials to manufacture PMUTs include aluminum nitride (AlN), lead zirconate titanate (PZT), and zinc oxide (ZnO). Some properties of them are shown in Table 1. The piezoelectric material is AlN in this work. Although the piezoelectric coefficient of AlN is lower than that of PZT and ZnO, it possesses high sensitivity because of its very low relative dielectric constant. What is more, AlN is compatible with standard CMOS technology, allowing monolithic integration of MEMS sensors. Additionally, the fabrication process of AlN is fully compatible with complementary metal-oxide-semiconductor (CMOS) fabrication process and is suitable for mass production [39].

As shown in Figure 1b, the PMUT array consists of four PMUTs (2 × 2) connected in parallel, each with an inner electrode (IE) and outer electrode (OE) on the top electrode layer. The fabrication process for PMUTs starts on a silicon on insulator (SOI) wafer. The thickness of device layer is 5 µm. The sandwich structure from bottom to top is bottom electrode Mo layer, AlN layer, and top electrode Mo layer by order, which are sputtered on the SOI wafer. Its schematic cross-sectional view is shown in Figure 1c. The corresponding geometric parameters of the PMUTs are summarized in Table 2. The size of PMUT array is 3.8 mm × 4.4 mm. The manufacturing process was completed at Shanghai Industrial µTechnology Research Institute (SITRI). Figure 1d shows the first mode shape of a single sensing diaphragm of the PMUT array simulated in COMSOL Multiphysics.

### 2.2. Characterization of the PMUT Array

**Figure 2:**
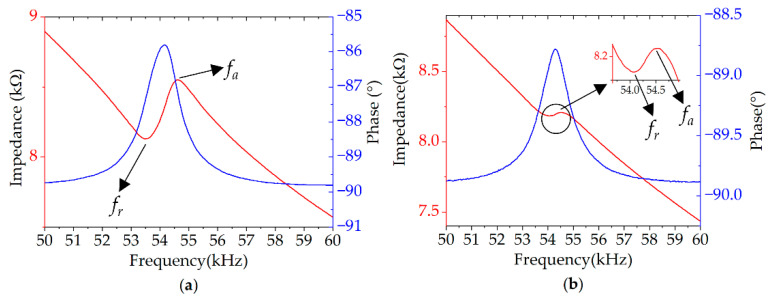
The impedance curves of the PMUT array of (**a**) only inner top electrodes and (**b**) only outer top electrodes.

The electrical impedance and amplitude–frequency characteristics of the PMUT array are measured. Figure 2 shows the electrical characterization results of the PMUT array measured through the impedance analyzer (Keysight E4990A, Beijing, China). The electrical resonant frequencies are measured to be 54.16 kHz, 54.27 kHz in the case of inner top electrodes and outer top electrodes, respectively. The resonance frequency measured under the two cases is similar. In the condition of the inner top electrodes, the resonant (*f_r_*) and anti-resonant (*f_a_*) frequencies are 53.48 kHz and 54.61 kHz, respectively. The resonant (*f_r_*) and anti-resonant (*f_a_*) frequencies of the outer top electrodes are 54.05 kHz and 54.53 kHz, respectively. The electromechanical coefficient of the inner top electrodes and outer top electrodes are 5% and 2%, respectively, according to Equation (1) [42]:

(1) $${kt}^{2}=\frac{\pi^{2}}{8}\frac{{fa}^{2}-{fr}^{2}}{{fa}^{2}}$$

**Figure 3:**
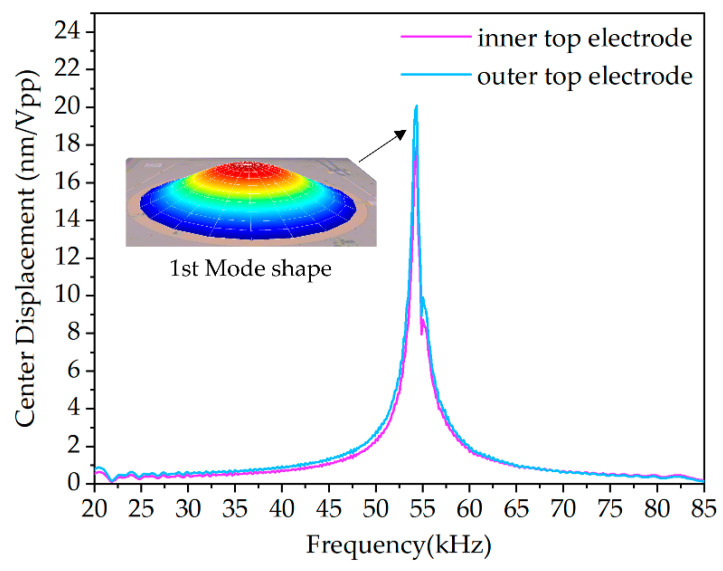
The measured frequency response of a sensing diaphragm in the PMUT array using LDV.

Figure 3 shows the curves of amplitude–frequency response of the inner top electrode and outer top electrode of a sensing diaphragm in the PMUT array using a laser Doppler vibrometer (LDV, Polytec UHF-120, Irvine, CA, USA) as well as the first mode shape (at the resonant frequency). According to the measurement results, the first resonance frequency of the sensing diaphragm is about 54 kHz and the displacement sensitivity of the center point of the inner top electrode and outer top electrode are 17.97 nm/Vpp and 20.08 nm/Vpp, respectively. The signal conditioning circuit with the three-electrodes form using two top electrodes and the bottom electrode is relatively complex in terms of circuit structure. At the same time, more complex processing programs in software are required when adopting the three-electrode form. Moreover, the resonant frequency and displacement sensitivity of a single top electrode are similar, so the inner electrodes are chosen in the selection of the top electrode.

## 3. Design of Sensing Module and Monitoring System

### 3.1. Sensing Module Design and Characterization

**Figure 4:**
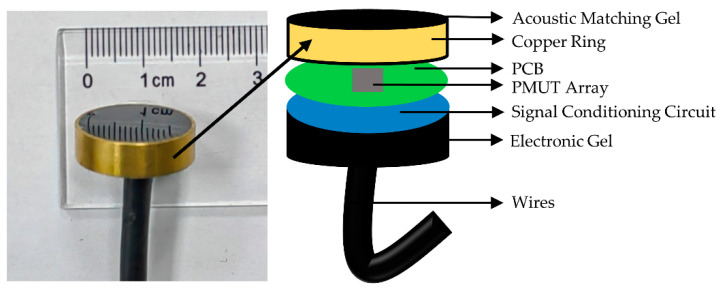
The packaged sensing module and structural diagram of the packaged sensing module.

**Figure 5:**
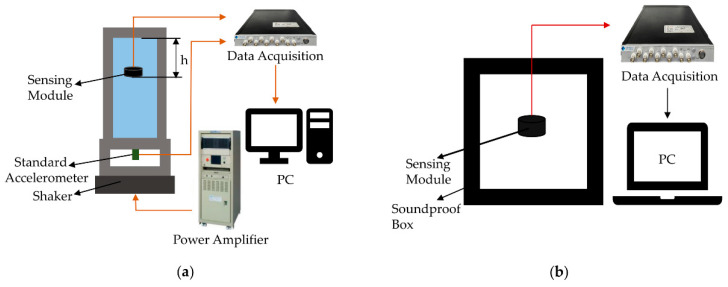
(**a**) Vibrating liquid column measurement setup. (**b**) Noise measurement setup.

**Figure 6:**
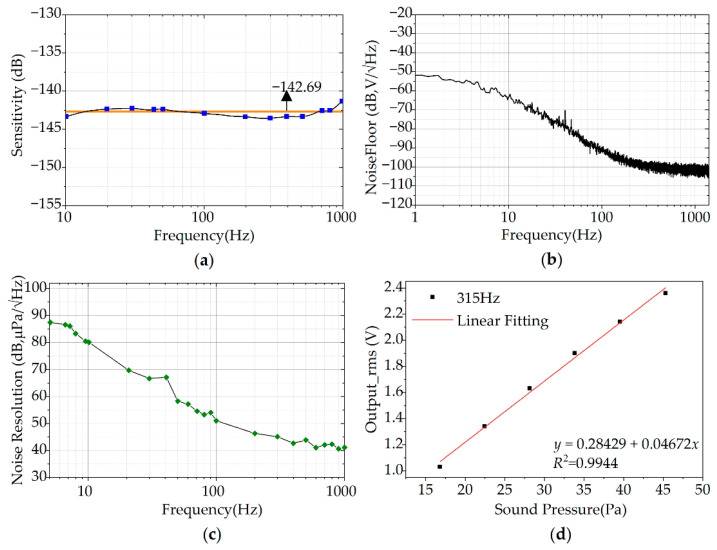
(**a**) The measured sensitivities of the sensing module. (**b**) The measured noise floor of the sensing module. (**c**) The calculated noise resolution of the sensing module. (**d**) Non-linearity measurement obtained by calculating the sound pressure at 315 Hz.

The sensing module consists of the PMUT array, a signal conditioning circuit, a metal housing, the acoustic matching gel, and the electronic gel. The structure from top to bottom is the acoustic matching gel, the PMUT array, a signal conditioning circuit, and the electronic gel. The charges generated by the PMUT array need to be converted into a suitable output signal through the signal conditioning circuit. The main functions of the signal conditioning circuit are signal amplification and impedance conversion. Since the amount of charge generated by a single PMUT array is very small, an array of four PMUTs are connected in parallel. As a result, the output charge of the array of four PMUTs connected in parallel is about four times higher than that of a single PMUT array, which can improve the sensitivity [43,44]. The front and back of the module are packaged with acoustic matching gel and electronic gel, respectively. The acoustic impedance of the average human soft tissue is about 1.63 MRayl [45]. To achieve acoustic impedance matching, the acoustic impedance of acoustic matching gel should be as close as possible to it. The applied acoustic matching gel is polyurethane, and its acoustic impedance is 1.50 MRayl, which is close to the average human soft tissue. The transmission coefficient of sound at the interface of the two media can be expressed as [46]:

(2) $$T=\frac{4Z_{1}Z_{2}}{{\left(Z_{2}+Z_{1}\right)}^{2}}$$

where *Z*_1_ and *Z*_2_ are the acoustic impedances (in MRayl) of two media. The transmission coefficient is 0.99 in this work. The packaged sensing module and structural diagram are shown in Figure 4. The diameter of the packaged module is 22 mm and the height is 6 mm. The sensitivity characterization of the sensing module is performed through the vibrating liquid column method [47]. The setup for sensitivity characterization is shown in Figure 5a. Firstly, the module is placed in the liquid column with water, and then *h* is recorded at this point. When measuring, the acceleration of the shaker is set to 0.05 g. The sensitivity of the sensing module is calculated according to Formula (3) [47]:

(3) $$S_{M}=\frac{U_{M}}{U_{a}}\times \frac{S_{a}}{\rho h}$$

where *U_M_*, *U_a_* are the output voltage of the sensing module and standard accelerometer, respectively. *S_a_* is the sensitivity of the accelerometer, *h* is the distance between the sensing module and the liquid level,ρ is the density of liquid. As is shown in Figure 6a, the result shows a very flat response over the operating frequency (10 Hz to 1 kHz), and the sensitivity of the sensing module is −142.69 dB (re: 1 V/μPa).

As a sensor for collecting weak bowel sounds, noise resolution is a very critical parameter. While the signals are enhanced using a signal conditioning circuit, the noise is also amplified together. The applied amplifier has noise voltage density of 4 nV/√Hz (at 1 kHz) and noise current density of 2.2 fA/√Hz (at 1 kHz). The noise resolution of the sensing module is calculated according to the Formula (4) [48]:

(4) $$\mathrm{NoiseResolution}=\frac{\mathrm{Noisefloor}}{\mathrm{Sensitivity}}$$

Noise floor is measured through the setup in Figure 5b. The whole measurement is performed in a soundproof box. Figure 6b shows the measured noise floor result of the sensing module. The calculated noise resolution of the sensing module is showed in Figure 6c. The sensing module has high output noise resolution of 80 dB, 50 dB, and 41 dB (re: 1 μPa/√Hz) at 10 Hz, 100 Hz, and 1 kHz, respectively. The high noise resolution benefits the collection of bowel sounds.

The small non-linearity of the sensing module can pick up a lot of acoustic signals from the human body [49]. The measured output voltage in relation to the sound pressure is shown in Figure 6d. In the measurement, a standard hydrophone is used to calibrate the sound pressure. The sound pressure increases from 16 Pa to 45 Pa at 315 Hz. At 315 Hz, the sensitivity of the standard hydrophone is flatter. The maximum non-linearity is about 0.1%.

### 3.2. Monitoring System Design

**Figure 7:**
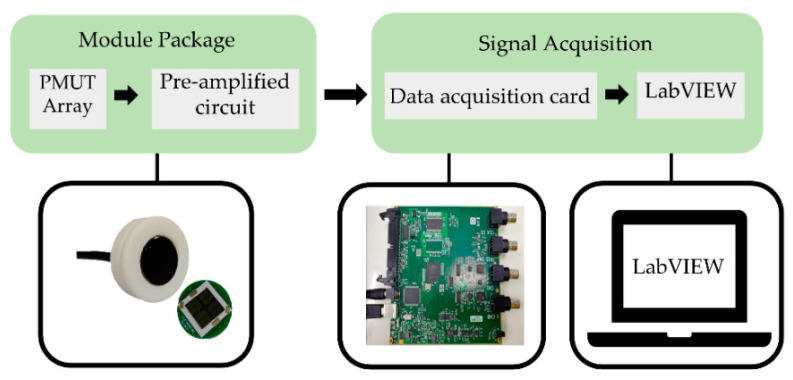
The conceptual representation of the developed PMUT array-based bowel sound monitor prototype in this work.

The monitoring system consists of the hardware system and the software system, which can achieve signal collection, transmission, and display in real time of bowel sounds. The hardware system mainly includes the sensing module, the data acquisition card, and a personal computer (PC). The software system is used to display in real time of bowel sounds. A fourth order Butterworth bandpass filter (bandpass frequency between 100 Hz and 1200 Hz) is used to process the collected data in the software. The sensing module is placed at a suitable position in the lower right quadrant of the human abdomen and is tightly attached to the human abdomen using a belt. The sensing module is powered by a power supply with a voltage of 12 V. The collected bowel sounds are sent to PC (LabVIEW) through the data acquisition card (MCC USB-2020, Shanghai, China). Subsequently, the PC (LabVIEW) receives the bowel sounds and displays the signal curves in real time. A schematic diagram of the bowel sound monitoring system is shown in Figure 7. Additionally, in order to keep the sensing module fixed to the belt better, this work uses 3D printing to design the support housing (the white part of the physical picture of the packaged module in the Figure 7), which has a diameter of about 35 mm.

## 4. Experimental Results and Discussion

### 4.1. Tissue-Mimicking Material Propagation Attenuation Experiment Based on Silicone

**Figure 8:**
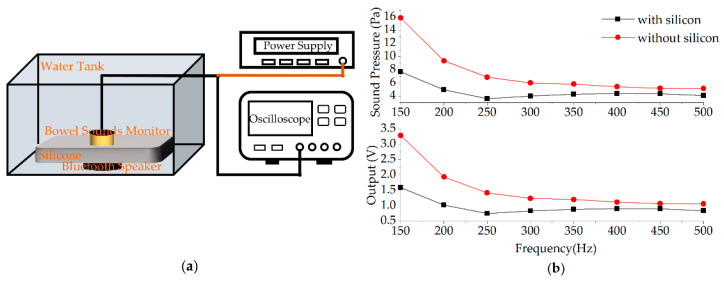
(**a**) Tissue-mimicking material propagation attenuation experiment setup. (**b**) The result of abdominal simulation material experiment.

Since bowel sounds are spread through the intestinal wall to the abdominal wall, they inevitably produce attenuation in the propagation. The purpose of this experiment is to simply explore the attenuation of sound pressure in body tissue. Tissue-mimicking materials are used to mimic soft tissue under certain conditions [50]. In this experiment, silicone is used to simulate the human abdomen and a water environment is used to simulate the environment inside human abdomen. The sounds from the waterproof Bluetooth speaker act as the sound source in the intestine. The experimental setup is shown in Figure 8a. The Bluetooth speaker could produce 150–500 Hz sine sound signals (sound volume is the same). The thickness of silicone is 2.34 cm, which is used to simulate the thickness of the human abdomen. The bowel sound monitor is placed on the upper surface of the silicone. The experiment is performed in the condition with silicone firstly and in the absence of silicone later. Figure 8b shows the result of the tissue-mimicking material experiment. The voltage obtained from the sensing module is converted into sound pressure. As shown in the results, 150–500 Hz frequencies have different attenuation in silicone, and the sound pressure is attenuated to varying extents.

### 4.2. Bowel Sound Monitoring Experiment

**Figure 9:**
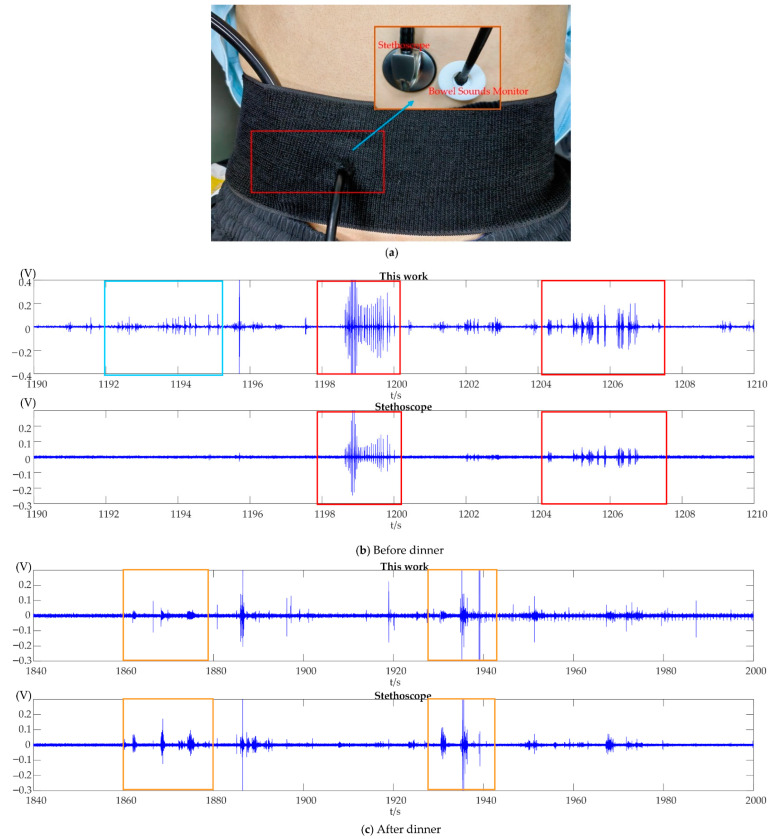
Bowel sound monitoring experiment. (**a**) Bowel sound measurement setup. (**b**) Bowel sounds obtained with a bowel sound monitor and a modified stethoscope before dinner. (**c**) Bowel sounds obtained with a bowel sound detector and a modified stethoscope after dinner.

To demonstrate the feasibility of the designed monitor, a modified stethoscope is used for comparison with it. The modified stethoscope is composed of an electret microphone and a stethoscope (the electret microphone is placed inside the rubber tube of the stethoscope). The volunteer is a healthy adult male (25 years old). The experiment is begun for half an hour before the meal and continues after the volunteer rests for 15 min after the meal, lasting for one and a half hours. Experimental data are collected in sets of 4 min each. The designed bowel sound monitor is placed on the lower right side of the abdomen, and the modified stethoscope is placed side by side with it, both as close as possible (as is shown in Figure 9a). Coupling gel is applied to the surface of the designed bowel sound monitor, which is used to reduce the signal propagation loss between different media.

**Figure 10:**
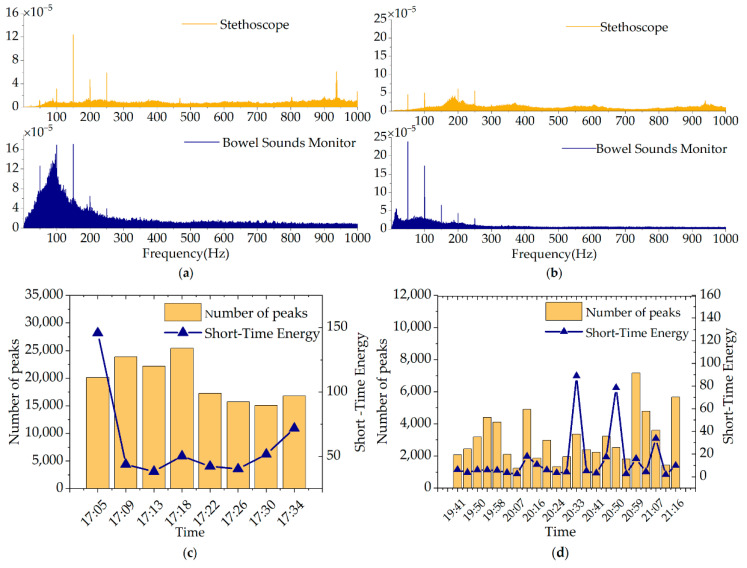
The spectrum of bowel sounds collected using the designed monitor (**a**) before dinner and (**b**) after dinner. Variation of number of peaks (in histogram) and short-time energy (broken line) of bowel sounds over time (**c**) before dinner and (**d**) after dinner using the designed monitor.

Segments of the time domain signals of bowel sounds collected from a healthy volunteer using the reported PMUT array-based bowel sound monitor and a modified stethoscope are shown in Figure 9b,c. There are some differences in the bowel sounds detected by the two methods. The reason is that the sensitivity of the reported monitor is higher than that of the stethoscope, and the designed bowel sound monitor can collect more signals. It is necessary to point out that the time–domain characteristics of the bowel sounds enclosed in blue boxes, red boxes, and orange boxes are very comparable to those of the single-burst, multiple-burst, and random continued sounds proposed by Du et al. [22]. According to the experimental results, features of the signals collected by the reported bowel sound monitor and the modified stethoscope are very similar. Moreover, the signals collected by it bear a strong resemblance to the study of Dimoulas et al. [51] on the bowel sounds in the time domain. Figure 10a,b represent the spectrum of bowel sounds obtained from the before-meal experiment and the after-meal experiment. The frequency of bowel sounds is mainly focused in the 100–500 Hz band, as is mentioned in the study [9]. Figure 10c,d show the variation the number of peaks and short-time energy over time. The number of peaks and short-time energy indicate the sum of the peaks and the energy within a period of 4 min, respectively. The number of peaks is used to describe the number of times the bowel sounds occur in a time period. Short-time energy reflects the amount of energy of the signal over a period of time. As can be seen from Figure 10c,d, intestinal activities are at a relatively high level before the meal (hunger state) and stay relatively stable for a period of time after the meal (satiety state) in this experiment. In conclusion, bowel sounds change a lot in a state of hunger and satiety through long-time monitoring. Some small and weak bowel sounds are not easily collected by a bulky stethoscope, but the designed monitor in this work can pick them up due to its high sensitivity. These small changes may carry some critical information about bowel sounds. In future work, we will explore the link between bowel sound signals and intestinal diseases.

## 5. Conclusions and Future Work

In this work, a prototype of a high-sensitivity and wearable bowel sound monitor based on PMUT array is designed for continuous monitoring of bowel sounds. The reported bowel sound monitor mainly consists of the sensing module to collect bowel sounds and a GUI based on LabVIEW to display bowel sound signals in real time. The sensing module is mainly composed of four parallel-connected PMUTs and a signal conditioning circuit. This work takes some performance measurements on the sensing module, and the result demonstrates that it possesses high sensitivity (−142.69 dB), high noise resolution (50 dB at 100 Hz), small non-linearity, and compact size. Furthermore, this work simply conducts an experiment on the attenuation of sound propagation in tissue-mimicking material first. The experimental result displays the sound attenuation in tissue-mimicking materials, which helps to understand the attenuation of sound in human tissue. Then, a long-time monitoring experiment was performed in a healthy male volunteer before and after dinner using the reported monitor and the modified stethoscope. In the monitoring experiment, some small and weak bowel sounds can be picked by the designed monitor due to its good performance, especially its high sensitivity, and they are not easily collected by the modified stethoscope. It is important to obtain weak bowel sound signals in daily intestinal health monitoring because it may help to explain the link between bowel sounds and intestinal diseases. The experimental results show that the PMUT array-based bowel sound monitor can achieve continuous monitoring of bowel sounds and shows good performance in collecting bowel sounds, which establishes the foundation for daily intestinal health monitoring in the future. In addition, the continuous monitoring experiment setup has limitations in that it was not performed on many volunteers (healthy people and patients). What is more, this work uses the inner top electrodes of the PMUT array and does not adopt the three-electrode form for the sake of simplifying the circuit and software programs. In the future, we will continue to explore the three-electrode form of the sensor as well as improve the experimental setup.

## Figures and Tables

**Table 1:** Properties of commonly used piezo materials [23,40,41].

Material	PZT	ZnO	AlN
Piezoelectric constant d33 (pC/N)	60–223	5.9–12.4	3.4–6.4
Dielectric constant	300–1300	10.9	8.5–10.5
Electromechanical coefficient k_t_^2^ (%)	7–15	9	6.5
tanδ (at 1–10 kHz, 10^5^ Vm^−1^)	0.01–0.03	0.01–0.1	0.003

**Table 2:** Geometric parameters of the PMUT array.

Material	Inner Top Mo	Outer Top Mo	AlN	Bottom Mo	Si	Cavity
Radius (µm)	540	780	-	-	-	800
Thickness (µm)	0.2	0.2	1	0.2	5	400
Width (µm)	-	220	-	-	-	-

## Data Availability

Not applicable.

## References

[1] Yoshino H., Abe Y., Yoshino T., Ohsato K. (1990). Clinical Application of Spectral Analysis of Bowel Sounds in Intestinal Obstruction. Dis. Colon Rectum.

[2] Hadjileontiadis L.J., Liatsos C.N., Mavrogiannis C.C., Rokkas T.A., Panas S.M. (2000). Enhancement of Bowel Sounds by Wavelet-Based Filtering. IEEE Trans. Biomed. Eng..

[3] Craine B.L., Silpa M.L., O’Toole C.J. (2001). Enterotachogram Analysis to Distinguish Irritable Bowel Syndrome from Crohn’s Disease. Dig. Dis. Sci..

[4] Liatsos C., Hadjileontiadis L.J., Mavrogiannis C., Patch D., Panas S.M., Burroughs A.K. (2003). Bowel Sounds Analysis: A Novel Noninvasive Method for Diagnosis of Small-Volume Ascites. Dig. Dis. Sci..

[5] Saito S.-N., Otsuka S., Zenbutsu S., Hori S., Honda M., Nakagawa S. Generation Mechanisms of Bowel Sounds by Simultaneous Measurements of X-Ray Fluoroscopy and Bowel Sounds. Proceedings of the 2021 43rd Annual International Conference of the IEEE Engineering in Medicine & Biology Society (EMBC).

[6] Baid H. (2009). A Critical Review of Auscultating Bowel Sounds. Br. J. Nurs..

[7] Cannon W.B. (1905). Auscultation of the Rhythmic Sounds Produced by the Stomach and Intestines. Am. J. Physiol. Leg. Content.

[8] Chanu O.R., Raj V.K. (2018). Acquisition and Characterization of Bowel Sounds Using LabVIEW Software. Biomed. Eng. Appl. Basis Commun..

[9] Ranta R., Louis-Dorr V., Heinrich C., Wolf D., Guillemin F. (2010). Digestive Activity Evaluation by Multichannel Abdominal Sounds Analysis. IEEE Trans. Biomed. Eng..

[10] Dalle D., Devroede G., Thibault R., Perrault J. (1975). Computer Analysis of Bowel Sounds. Comput. Biol. Med..

[11] Goto J., Matsuda K., Harii N., Moriguchi T., Yanagisawa M., Sakata O. (2015). Usefulness of a Real-Time Bowel Sound Analysis System in Patients with Severe Sepsis (Pilot Study). J. Artif. Organs.

[12] Emoto T., Abeyratne U.R., Gojima Y., Nanba K., Sogabe M., Okahisa T., Akutagawa M., Konaka S., Kinouchi Y. (2016). Evaluation of Human Bowel Motility Using Non-Contact Microphones. Biomed. Phys. Eng. Express.

[13] Kolle K., Fougner A.L., Ellingsen R., Carlsen S.M., Stavdahl O. (2019). Feasibility of Early Meal Detection Based on Abdominal Sound. IEEE J. Transl. Eng. Health Med..

[14] Turk E., Oztas A.S., Deniz Ulusar U., Canpolat M., Kazanir S., Yaprak M., Ogunc G., Dogru V., Canagir O.C. Wireless bioacoustic sensor system for automatic detection of bowel sounds. Proceedings of the 2015 19th National Biomedical Engineering Meeting (BIYOMUT).

[15] Spiegel B.M.R., Kaneshiro M., Russell M.M., Lin A., Patel A., Tashjian V.C., Zegarski V., Singh D., Cohen S.E., Reid M.W. (2014). Validation of an Acoustic Gastrointestinal Surveillance Biosensor for Postoperative Ileus. J. Gastrointest. Surg..

[16] Garner C.G., Ehrenreich H. (1989). Non-Invasive Topographic Analysis of Intestinal Activity in Man on the Basis of Acustic Phenomena. Res. Exp. Med..

[17] Qiao Y., Wang L., Tao X. A Bowel Sound Detection Method Based on a Novel Non-Speech Body Sound Sensing Device. Proceedings of the 2021 IEEE 45th Annual Computers, Software, and Applications Conference (COMPSAC).

[18] Chien C.-H., Huang H.-T., Wang C.-Y., Chong F.-C. (2009). Two-Dimensional Static and Dynamic Display System of Bowel Sound Magnitude Map for Evaluation of Intestinal Motility. Biomed. Eng. Appl. Basis Commun..

[19] Hadjileontiadis L.J., Rekanos I.T. (2003). Detection of Explosive Lung and Bowel Sounds by Means of Fractal Dimension. IEEE Signal Process. Lett..

[20] Yin Y., Yang W., Jiang H., Wang Z. Bowel Sound Based Digestion State Recognition Using Artificial Neural Network. Proceedings of the 2015 IEEE Biomedical Circuits and Systems Conference (BioCAS).

[21] Horiyama K., Emoto T., Haraguchi T., Uebanso T., Naito Y., Gyobu T., Kanemoto K., Inobe J., Sano A., Akutagawa M. (2021). Bowel Sound-Based Features to Investigate the Effect of Coffee and Soda on Gastrointestinal Motility. Biomed. Signal Process. Control.

[22] Du X., Allwood G., Webberley K., Osseiran A., Marshall B. (2018). Bowel Sounds Identification and Migrating Motor Complex Detection with Low-Cost Piezoelectric Acoustic Sensing Device. Sensors.

[23] Jung J., Lee W., Kang W., Shin E., Ryu J., Choi H. (2017). Review of Piezoelectric Micromachined Ultrasonic Transducers and Their Applications. J. Micromech. Microeng..

[24] Przybyla R., Izyumin I., Kline M., Boser B., Shelton S. An Ultrasonic Rangefinder Based on an AlN Piezoelectric Micromachined Ultrasound Transducer. Proceedings of the 2010 IEEE Sensors.

[25] Przybyla R.J., Shelton S.E., Guedes A., Izyumin I.I., Kline M.H., Horsley D.A., Boser B.E. (2011). In-Air Rangefinding With an AlN Piezoelectric Micromachined Ultrasound Transducer. IEEE Sens. J..

[26] Muralt P., Baborowski J. (2004). Micromachined Ultrasonic Transducers and Acoustic Sensors Based on Piezoelectric Thin Films. J. Electroceram..

[27] Cai J., Wang Y., Lou L., Zhang S., Gu Y., Gao F., Wu T. Photoacoustic and Ultrosound Dual-Modality Endoscopic Imaging Based on ALN Pmut Array. Proceedings of the 2022 IEEE 35th International Conference on Micro Electro Mechanical Systems Conference (MEMS).

[28] Jiang L., Lu G., Zeng Y., Sun Y., Kang H., Burford J., Gong C., Humayun M.S., Chen Y., Zhou Q. (2022). Flexible Ultrasound-Induced Retinal Stimulating Piezo-Arrays for Biomimetic Visual Prostheses. Nat. Commun..

[29] Zeng Y., Jiang L., Sun Y., Yang Y., Quan Y., Wei S., Lu G., Li R., Rong J., Chen Y. (2020). 3D-Printing Piezoelectric Composite with Honeycomb Structure for Ultrasonic Devices. Micromachines.

[30] Liu C., Djuth F., Hu C., Chen R., Zhang X., Li X., Zhou Q., Shung K. (2012). Micromachined High Frequency PMN-PT/Epoxy 1-3 Composite Ultrasonic Annular Arrays. Ultrasonics.

[31] Zhou Q., Lau S., Wu D., Kirk Shung K. (2011). Piezoelectric Films for High Frequency Ultrasonic Transducers in Biomedical Applications. Prog. Mater. Sci..

[32] Wang F., Wu D., Jin P., Zhang Y., Yang Y., Ma Y., Yang A., Fu J., Feng X. (2019). A Flexible Skin-Mounted Wireless Acoustic Device for Bowel Sounds Monitoring and Evaluation. Sci. China Inf. Sci..

[33] Kodani K., Sakata O. Automatic Bowel Sound Detection under Cloth Rubbing Noise. Proceedings of the 2020 IEEE Region 10 Conference (TENCON).

[34] Sakata O., Matsuda K., Suzuki Y., Satake T. Basic Study of Occurrence Frequency of Bowel Sounds after Food Ingestion. Proceedings of the TENCON 2011—2011 IEEE Region 10 Conference.

[35] Shkel A.A., Kim E.S. (2019). Continuous Health Monitoring With Resonant-Microphone-Array-Based Wearable Stethoscope. IEEE Sens. J..

[36] Baronetto A., Graf L.S., Fischer S., Neurath M.F., Amft O. GastroDigitalShirt: A Smart Shirt for Digestion Acoustics Monitoring. Proceedings of the 2020 International Symposium on Wearable Computers.

[37] Allwood G., Du X., Webberley K.M., Osseiran A., Marshall B.J. (2019). Advances in Acoustic Signal Processing Techniques for Enhanced Bowel Sound Analysis. IEEE Rev. Biomed. Eng..

[38] Lu Y., Wang Q., Horsley D.A. Piezoelectric Micromachined Ultrasonic Transducers with Increased Coupling Coefficient via Series Transduction. Proceedings of the 2015 IEEE International Ultrasonics Symposium (IUS).

[39] Wang Q., Lu Y., Mishin S., Oshmyansky Y., Horsley D.A. (2017). Design, Fabrication, and Characterization of Scandium Aluminum Nitride-Based Piezoelectric Micromachined Ultrasonic Transducers. J. Microelectromech. Syst..

[40] Ali W.R., Prasad M. (2020). Piezoelectric MEMS Based Acoustic Sensors: A Review. Sens. Actuators A Phys..

[41] Tadigadapa S., Mateti K. (2009). Piezoelectric MEMS Sensors: State-of-the-Art and Perspectives. Meas. Sci. Technol..

[42] Luo G.-L., Kusano Y., Horsley D.A. (2021). Airborne Piezoelectric Micromachined Ultrasonic Transducers for Long-Range Detection. J. Microelectromech. Syst..

[43] Fang L.H., Rahim R.b.A., Romli M.I.F., Zakariya M.Z., Jobran J.B.A.M., Kimpol N.B. Piezoelectric Array Configuration Technique into Enhance Power Catchment for Sound Energy Harvester System. Proceedings of the 2020 International Conference on Sustainable Energy Engineering and Application (ICSEEA).

[44] Sun C., Shang G., Zhu X., Tao Y., Li Z. Modeling for Piezoelectric Stacks in Series and Parallel. Proceedings of the 2013 Third International Conference on Intelligent System Design and Engineering Applications.

[45] Culjat M.O., Goldenberg D., Tewari P., Singh R.S. (2010). A Review of Tissue Substitutes for Ultrasound Imaging. Ultrasound Med. Biol..

[46] Tiefensee F., Becker-Willinger C., Heppe G., Herbeck-Engel P., Jakob A. (2010). Nanocomposite Cerium Oxide Polymer Matching Layers with Adjustable Acoustic Impedance between 4 MRayl and 7 MRayl. Ultrasonics.

[47] Yang D., Yang L., Chen X., Qu M., Zhu K., Ding H., Li D., Bai Y., Ling J., Xu J. (2021). A Piezoelectric AlN MEMS Hydrophone with High Sensitivity and Low Noise Density. Sens. Actuators A Phys..

[48] Xu J., Zhang X., Fernando S.N., Chai K.T., Gu Y. (2016). AlN-on-SOI Platform-Based Micro-Machined Hydrophone. Appl. Phys. Lett..

[49] Jia L., Shi L., Liu C., Xu J., Gao Y., Sun C., Liu S., Wu G. (2022). Piezoelectric Micromachined Ultrasonic Transducer Array-Based Electronic Stethoscope for Internet of Medical Things. IEEE Internet Things J..

[50] Thouvenot A., Poepping T., Peters T.M., Chen E.C.S. Characterization of Various Tissue Mimicking Materials for Medical Ultrasound Imaging. Proceedings of the Medical Imaging 2016: Physics of Medical Imaging.

[51] Dimoulas C., Kalliris G., Papanikolaou G., Kalampakas A. (2007). Long-Term Signal Detection, Segmentation and Summarization Using Wavelets and Fractal Dimension: A Bioacoustics Application in Gastrointestinal-Motility Monitoring. Comput. Biol. Med..

